# Phytochemical Analysis of Phenolics, Sterols, and Terpenes in Colored Wheat Grains by Liquid Chromatography with Tandem Mass Spectrometry

**DOI:** 10.3390/molecules26185580

**Published:** 2021-09-14

**Authors:** Mayya P. Razgonova, Alexander M. Zakharenko, Elena I. Gordeeva, Olesya Yu. Shoeva, Elena V. Antonova, Konstantin S. Pikula, Liudmila A. Koval, Elena K. Khlestkina, Kirill S. Golokhvast

**Affiliations:** 1 N.I. Vavilov All-Russian Institute of Plant Genetic Resources, B. Morskaya 42-44, 190000 Saint Petersburg, Russia; zakharenko.am@dvfu.ru (A.M.Z.); elgordeeva@bionet.nsc.ru (E.I.G.); selena@ipae.uran.ru (E.V.A.); pikula_ks@dvfu.ru (K.S.P.); 2 Institute of Cytology and Genetics, Siberian Branch of Russian Academy of Sciences, Lavrentjeva 10, 630090 Novosibirsk, Russia; 3 Institute of Plant and Animal Ecology, Ural Branch of Russian Academy of Sciences, 8 Marta 202, 620144 Ekaterinburg, Russia; 4 School of Biomedicine, Far Eastern Federal University, Sukhanova 8, 690950 Vladivostok, Russia; koval.liudmila.an@mail.ru; 5 Pacific Geographical Institute, Far Eastern Branch of the Russian Academy of Sciences, Radio 7, 690041 Vladivostok, Russia; 6 Siberian Federal Scientific Centre of Agrobiotechnology, Centralnaya, Presidium, 633501 Krasnoobsk, Russia

**Keywords:** *Triticum aestivum*, colored wheat, anthocyanin, HPLC-MS/MS, tandem mass spectrometry, phenolic compound, biologically active compound

## Abstract

The colored grain of wheat (*Triticum aestivum* L.) contains a large number of polyphenolic compounds that are biologically active ingredients. The purpose of this work was a comparative metabolomic study of extracts from anthocyaninless (control), blue, and deep purple (referred to here as black) grains of seven genetically related wheat lines developed for the grain anthocyanin pigmentation trait. To identify target analytes in ethanol extracts, high-performance liquid chromatography was used in combination with Bruker Daltonics ion trap mass spectrometry. The results showed the presence of 125 biologically active compounds of a phenolic (85) and nonphenolic (40) nature in the grains of *T. aestivum* (seven lines). Among them, a number of phenolic compounds affiliated with anthocyanins, coumarins, dihydrochalcones, flavan-3-ols, flavanone, flavones, flavonols, hydroxybenzoic acids, hydroxycinnamic acids, isoflavone, lignans, other phenolic acids, stilbenes, and nonphenolic compounds affiliated with alkaloids, carboxylic acids, carotenoids, diterpenoids, essential amino acids, triterpenoids, sterols, nonessential amino acids, phytohormones, purines, and thromboxane receptor antagonists were found in *T. aestivum* grains for the first time. A comparative analysis of the diversity of the compounds revealed that the lines do not differ from each other in the proportion of phenolic (53.3% to 70.3% of the total number of identified compounds) and nonphenolic compounds (46.7% to 29.7%), but diversity of the compounds was significantly lower in grains of the control line. Even though the lines are genetically closely related and possess similar chemical profiles, some line-specific individual compounds were identified that constitute unique chemical fingerprints and allow to distinguish each line from the six others. Finally, the influence of the genotype on the chemical profiles of the wheat grains is discussed.

## 1. Introduction

**Figure 1:**
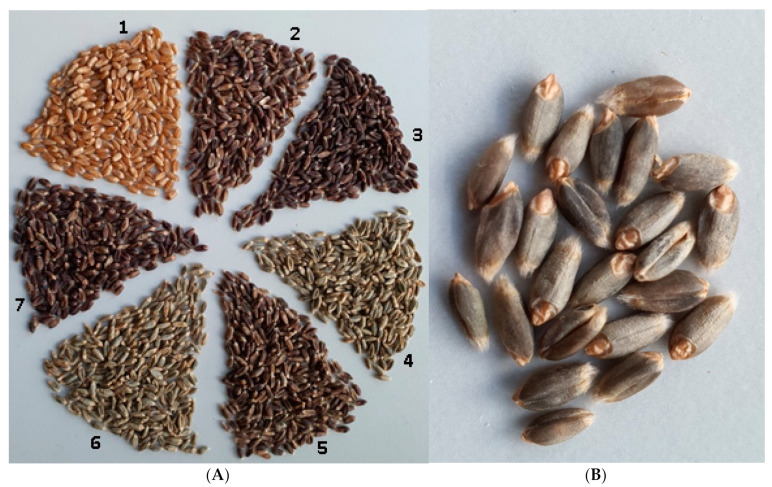
Diversity of colors among the analyzed wheat lines (having anthocyanin-rich grains). (**A**) The grains of the wheat lines used in this study: control line Saratovskaya 29 (S29) (1) in the upper left-hand corner and next in clockwise order S29 BLACK (4Th-4B) (2), S29 BLACK (4Th-4D) (3), S29 BLUE (4Th-4D) (4), BW BLACK (4Th-4D) (5), S29 BLUE (4Th-4B) (6), and E22 BLACK (4Th-4D) (7). (**B**) Grains of S29 BLUE (4Th-4B).

Among nutritional sources of antioxidant compounds necessary for human health, cereal products, which contain flavonoid pigments (plant compounds of a phenolic nature), are now receiving increasing attention [1]. The biosynthesis of various colored flavonoid compounds in certain grain components of cereal plants gives rise to a distinct color (Figure 1). As a result of the biosynthesis of anthocyanins, cereal seeds can have a color of various shades from bluish gray and reddish to dark purple and almost black. Other classes of flavonoid compounds give the grain a reddish-brown color (proanthocyanidins) or a dark-brown color (phlobaphenes). Anthocyanins have the highest antioxidant potential among the above-mentioned compounds [2]. These substances can accumulate in vegetative and reproductive parts of the plant, where their main physiological role is to protect the plant from excessive UV radiation. Additionally, the concentration of anthocyanins usually increases during exposure to adverse environmental factors [3].

Anthocyanins have been shown to play an important part in the prevention of neurodegenerative diseases [4], atherosclerosis, diabetes, and obesity and to have vasoprotective and anti-inflammatory properties [5,6]. As a consequence, the food industry is interested in researching colored cereals.

In the most common cereal species, soft wheat (*Triticum aestivum* L.), the grain can have either an unremarkable color or a reddish-brown, bluish-gray, or purple hue. The differences in color are due to the accumulation of certain flavonoid pigments in various layers of wheat grain envelopes [7,8,9]. The biosynthesis of proanthocyanidins in the seed coat causes a reddish-brown hue (trait: “red grain”) and is controlled by *R* genes localized on chromosomes of homoeologous group 3 [10]. The bluish-gray hue appears to be due to the biosynthesis of anthocyanins in the aleurone layer (trait: “blue aleurone”) and is regulated by *Ba* genes introduced into the common wheat genome from wild relatives such as wheatgrass *Thinopyrum ponticum*, *Triticum boeoticum*, and *Thinopyrum bessarabicum* owing to translocations in the chromosomes of homoeologous group 4 or via a substitution of one of the chromosomes of homoeologous group 4 [11,12,13]. The purple color is a consequence of the biosynthesis of anthocyanins in cells of the pericarp (trait: “purple pericarp”). This trait is regulated by two complementary genes, *Pp-1* and *Pp3*, mapped to chromosomes of homoeologous group 7 and chromosome 2A, respectively [8,14,15]. The inclusion of the genes that control the biosynthesis of anthocyanins in the grain into the breeding process may ultimately increase nutritional value of whole-grain products [1,16]. This notion has been demonstrated in end-use bakery products prepared from anthocyanin-rich wheat grains such as whole-grain bread [17,18], biscuits [16,19], pasta [20], pancakes, porridge, crackers, and candy bars [21]. The amount of anthocyanins in whole-grain blue-wheat bread and purple-wheat bread made according to a traditional Czech recipe has been determined, and how thermal parameters, such as temperature and baking time, affect individual anthocyanins and the total amount of anthocyanins in bread has been shown [18].

The predominant anthocyanin in purple and blue wheat varieties is cyanidin-3-glycoside, and each wheat variety is reported to have a specific anthocyanin profile [22]. Thirteen anthocyanins have been identified in purple wheat, among which cyanidin-3-glucoside is the most abundant, followed by cyanidin-3-galactoside and malvidin-3-glucoside. In purple wheat, anthocyanins are also present in the form of pelargonidin-3-glucoside and anthocyanins glycosylated with arabinose [23]. Besides anthocyanins, other bioactive phytochemicals such as phenolic acids, carotenoids, tocopherols, and phytosterols can be found in the wheat grain [24], and determination of their profiles in colored wheat grains will allow to take full practical advantage of colored wheat varieties. Therefore, the aim of this study was to identify anthocyanins and the total polyphenolic profile along with accompanying sterols and stilbenes in seven wheat lines having grains of different colors by high-performance liquid chromatography (HPLC) coupled with tandem mass spectrometry (MS/MS) on an ion trap instrument. The lines have been previously developed for the grain anthocyanin pigmentation trait in a common genetic background of anthocyaninless cultivar Saratovskaya 29 (S29) (four lines) used here as a control and in the genetic background of the breeding lines promising in terms of cultivation in Western Siberia (two lines). Two blue-grained lines—S29 BLUE (4Th-4B) and S29 BLUE (4Th-4D)—are substitution lines where chromosomes 4B and 4D are substituted with *Th. ponticum* chromosome 4Th carrying the *Ba* gene determining the blue pigmentation of grains [25,26]. Two deep-purple-grained, i.e., almost black-grained lines—S29 BLACK (4Th-4B) and S29 BLACK (4Th-4D)—in addition to chromosomes 4B and 4D substituted with 4Th, feature introgressions in chromosomes 7D and 2A, where the dominant alleles of genes *Pp-D1* and *Pp3*, respectively, are located [25]. Two other black-grained lines—BW BLACK (4Th-4D) and E22 BLACK (4Th-4D)—were developed in the genetic background of breeding line BW49880 (BW) and *cv.* Element 22 (E22), respectively, by means of S29 sister lines as donors of genes *Pp* and *Ba* in the current study. The use of such genetically related lines in a metabolomic study should enable us to draw conclusions about the effects of chromosome substitutions and introgression fragments on the chemical profile of the grains. To our knowledge, this is the first extensive study on anthocyanins and related polyphenols in unpigmented, blue-grained, and purple-grained wheat lines.

## 2. Results

### 2.1. Chemical Identification of the Wheat Grain Metabolites

**Figure 2:**
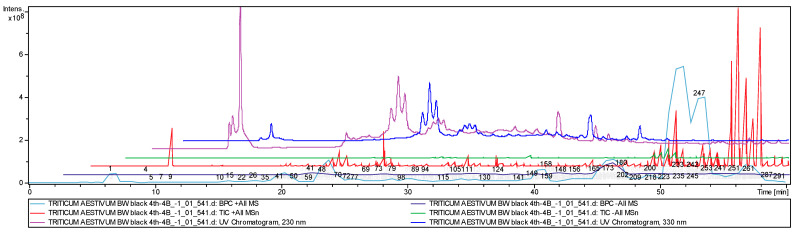
Chemical profiles of the BW BLACK (4Th-4D) sample presented as a total ion chromatogram from the EtOH extract.

A total of 300 peaks were detected in the chromatogram (Figure 2). After a comparison of the *m*/*z* values, retention times, and the fragmentation patterns with the MS/MS spectral data retrieved from the cited articles and after a database search (MS2T, MassBank, HMDB), a comprehensive table was compiled of the molecular masses of the analytes of interest isolated from ethanolic extracts of *T. aestivum* grains for ease of annotation (Appendix A). The 125 identified biologically active compounds are presented in Table 1. Among them, 85 compounds belong to various polyphenolic families: anthocyanins, flavones, flavonols, flavan-3-ols, flavanones, hydroxycinnamic acids, hydroxybenzoic acids, stilbenes, and coumarins, and the other 40 compounds are nonphenolic substances. In addition to previously reported metabolites, a number of metabolites were found for the first time in *T. aestivum* grains. Among them, there were anthocyanins (cyanidin 3-(2″-galloylglucoside) and petunidin), coumarins (fraxetin and fraxetin-7-*O*-sulfate), dihydrochalcones (phlorizin), flavan-3-ols (epicatechin and gallocatechin), flavanone (naringenin), flavones (acacetin *C*-glucoside methylmalonylated, apigenin, apigenin 6-*C*-deoxyhexoside-8-*C*-pentoside, dihydroxy tetramethoxyflavanone, cirsiliol, genistein *C*-glucosylglucoside, hydroxy dimethoxyflavone hexoside, myricetin, orientin 7-*O*-deoxyhexoside, pentahydroxy dimethoxyflavone, pentahydroxy dimethoxyflavone hexoside, pentahydroxy trimethoxy flavone, tetrahydroxy-dimethoxyflavone-hexoside, trihydroxy methoxyflavone triacetate, and vitexin 6″-*O*-glucoside), flavonols (ampelopsin, isorhamnetin, kaempferide, kaempferol, rhamnetin I, rhamnetin II, taxifolin-3-*O*-glucoside, and taxifolin-*O*-pentoside), hydroxybenzoic acids (*cis*-salvianolic acid J, gallic acid hexoside, hydroxy methoxy dimethylbenzoic acid, salvianolic acid D, salvianolic acid F, and salvianolic acid G), hydroxycinnamic acids (1-caffeoyl-β-d-glucose, 1-*O*-Sinapoyl-β-d-glucose, caffeic acid derivative, caftaric acid, and ferulic acid methyl ester), isoflavone (wighteone-*O*-glucoside), lignans (dimethyl-secoisolariciresinol, podophyllotoxin), other phenolic acids (1-*O*-caffeoyl-5-*O*-feruloylquinic acid, 4-*O*-Caffeoyl-5-*O*-*p*-coumaroylquinic acid, and feruloyl sulfate), stilbenes (pinosylvin, polydatin, and resveratrol), alkaloids (berberine and sespendole), carboxylic acids (9,10-dihydroxy-8-oxooctadec-12-enoic acid, 11-hydroperoxy-octadecatrienoic acid, dihydroxy docosanoic acid, docosenoic acid, myristoleic acid, pentacosenoic acid, salvianic acid C, and undecanedioic acid), carotenoids (cryptoxanthin and (*3S*, 3′*S*, all-*E*)-zeaxanthin), diterpenoids (isocryptotanshinone II and tanshinone IIB), essential amino acids (l-histidine, l-tryptophan, and l-valine), triterpenoids (β-amyrin, squalene, uvaol, and ursolic acid), sterols (avenasterol, brassicasterol, β-sitostenone, β-sitosterin, campestenone, ergosterol, fucosterol, oxo-hydroxy sitosterol, vebonol), steroids (cyclopassifloic acid glucoside), nonessential amino acids (tyrosine), phytohormone (GA8-hexose gibberellin), propionic acid (ketoprofen), purine (adenosine), and thromboxane receptor antagonist (vapiprost).

The flavone family featured the greatest number of members (32 substances) among the analyzed wheat grains; the flavonol family (10), anthocyanins (10), cinnamic acids (seven), lignin (five), and hydroxybenzoic acids (six) were found much less frequently. Among other identified compounds, i.e., nonphenolic substances, sterols (six compounds), higher-molecular-weight carboxylic acids (seven), and di- and triterpenoids (eight) were detected most often.

### 2.2. Similarities and Differences in Metabolites among the Lines

**Figure 3:**
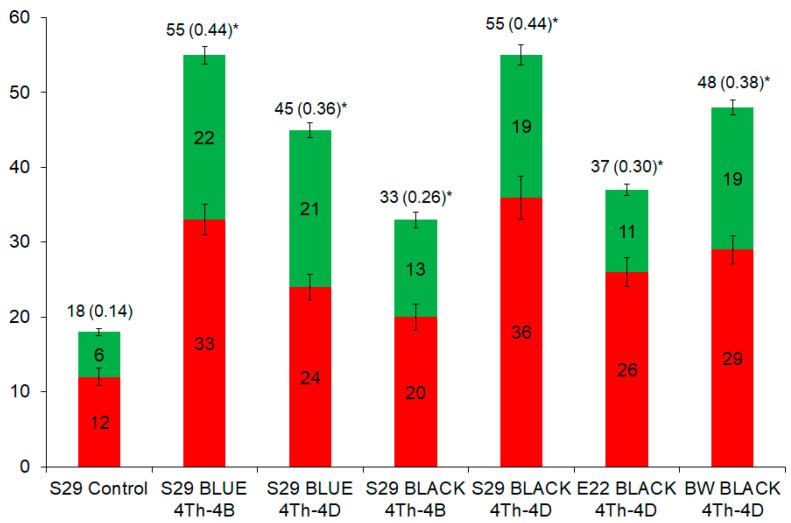
The number of the phenolic (red) and non-phenolic (green) compounds that were detected in the differently colored grains of the seven wheat lines. Error bars denote standard deviation; the number of individual compounds and its proportion among all the annotated compounds (125) are shown above the bars. * A significant difference from the control line.

According to Table 1 and Figure 3, the largest number of biologically active compounds (55) was found in lines S29 BLUE (4Th-4B) and S29 BLACK (4Th-4D), and the smallest (18) in the control line (differences of S29 from all the other lines are significant according to a two-sided test for proportions [Spearman’s rank correlation analysis], *p* = 0.00001–0.0177). Similar data were obtained for the polyphenol family: the largest (33 and 36) and smallest (12) numbers of such compounds were detected in the same lines as mentioned above. Phenolic compounds were found more often than nonphenolic compounds (*p* = 0.00001–0.0016) in all studied lines except for S29 BLUE (4Th-4D). In this line, the two classes of compounds showed almost equal numbers of members (*p* = 0.3428). Overall, in terms of the numbers of substances of a phenolic nature (53.3–70.3% of the total number of identified compounds) and a nonphenolic nature (46.7–29.7%) the studied lines were similar.

**Figure 4:**
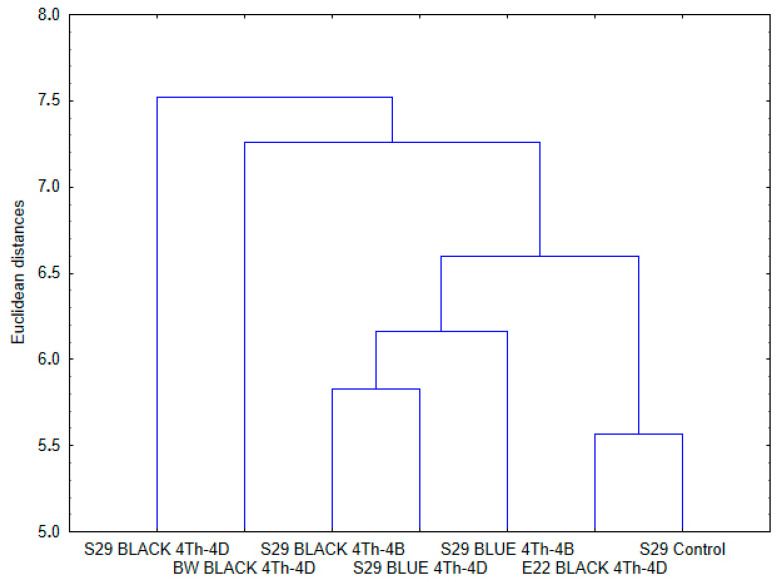
This tree was constructed by the unweighted pair group method with arithmetic mean (UPGMA) (based on Euclidean distances) from the data on 125 phenolic and nonphenolic substances of the seven *T. aestivum* lines.

The results of cluster analysis of all the compounds (Figure 4) showed that two clusters can be distinguished in the dendrogram. The first cluster is formed by lines S29 BLACK (4Th-4B) and S29 BLUE (4Th-4D) and the adjacent S29 BLUE (4Th-4B) line. The second cluster consists of the control line S29 and of E22 BLACK (4Th-4D). Lines BW BLACK (4Th-4D) and S29 BLACK (4Th-4D) did not end up in any clusters. Analysis of Spearman’s rank correlations confirmed the results of the cluster analysis. It was found that pairs of isogenic lines “S29 BLUE (4Th-4B)/S29 BLUE (4Th-4D)” and “S29 BLUE (4Th-4B)/S29 BLACK (4Th-4B)” (located in one cluster) are close to each other (*R*_S_ = 0.346–0.409, *p* < 0.05). Similar results were obtained on the second cluster in the “S29/E22 BLACK (4Th-4D)” pair (*R*_S_ = 0.333, *p* < 0.05). In addition, statistically significant correlation coefficients (*R*_S_ = 0.243–0.287, *p* < 0.05) were obtained in the comparison of the pair of lines with a substituted 4D chromosome “S29 BLUE (4Th-4D)/E22 BLACK (4Th-4D)” and a pair of lines with the black seed color “S29 BLACK (4Th-4B)/E22 BLACK (4Th-4D)”.

Plotting of dendrograms separately for phenolic and nonphenolic families of substances indicated that nonphenolic compounds differentiate lines by grain color (Figure S1). Even clearer separation by grain color was noted when the lignin family of compounds was utilized for the tree construction. Similar data were obtained on anthocyanins, flavones, and terpenoids. Unambiguous separation by substituted chromosomes was not achieved by means of any one family of substances. In some cases (e.g., for sterols and flavonols), one cluster was distinguished on the basis of the seed color, and the other cluster on the basis of chromosome substitution (Figure S1).

Examination of the chemical composition of wheat grains by the families of compounds within the phenolic and nonphenolic classes revealed that the lower number of biologically active substances detected in the control line can be explained by the absence of seven families of phenolic substances: coumarins, flavan-3-ols, flavanones, flavonols, phenolic acids, dihydrochalcone, and stilbenes. Flavonols were found in all the lines except for the control (S29). Furthermore, in S29, the number of substances belonging to the most numerous (in this study) “flavones” was 1.4–3.2-fold lower as compared to the other lines. The lower number of nonphenolic substances detected in the control line can be explained by the absence of the following families: alkaloids, anabolic steroids, carboxylic acids, carotenoids, cycloartanols, di- and triterpenoids, propionic acids, purines, sesquiterpenoid plant hormones, thromboxane receptor antagonists, and unsaturated fatty acids. Accordingly, the colored-grain lines showed a 3–6-fold greater number of substances in the carboxylic acid family, 2–3-fold in the sterol family, 2–4-fold in the anthocyanin family, and 1.5–2.7-fold in the flavone family as compared to the unpigmented-grain control (S29). It should also be noted that among the phenolic compounds, selgin (from the flavonol family) and abscisic acid [dormin; abscisin II; (*S*)−(+)-abscisic acid] from the class of nonphenolic compounds (sesquiterpenoid plant hormone family) were found only in lines with a substitution of chromosome 4B. A number of compounds (peonidin-3-*O*-glucoside, caffeic acid derivative, apigenin, isorhamnetin, kaempferol, rhamnetin II, taxifolin-*O*-pentoside, salvianolic acid G, undecanedioic acid, cyclopassifloic acid glucoside, sespendole, berberine, and β-sitostenone) were found only in some lines with a substituted 4D chromosome (Table S1, 13 substances in total). In addition, some detected substances proved to be characteristic of only wheat with blue grains (malvidin 3-*O*-rutinoside-5-*O*-glucoside, petunidin 3-*O*-rutinoside-5-*O*-glucoside, apigenin 2″-*O*-sinapoyl, *C*-hexosyl, *C*-pentosyl, vicenin-2, isocryptotanshinone II, and vapiprost) or black grains (isorhamnetin and taxifolin-*O*-pentoside). The latter case includes only the lines with a substitution of chromosome 4D.

**Figure 5:**
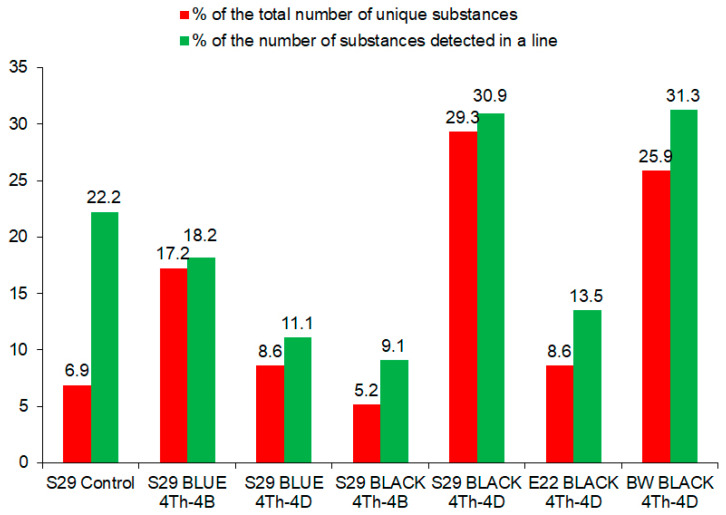
Assessment of the contributions of unique substances to the total pool of phenolic and nonphenolic compounds in the seven lines of *T. aestivum*.

Among the 125 compounds identified in this study in wheat grains, 58 substances turned out to be unique, that is, each was detectable in only one of the seven analyzed lines. The lowest number of unique compounds (three and four) was found in lines S29 BLACK (4Th-4B) and S29, respectively, and the highest number (17 and 15) in S29 BLACK (4Th-4D) and BW BLACK (4Th-4D). The rest of the lines were somewhere in between. These data are in good agreement with the contribution of the unique compounds to the total pool of detected substances (Figure 5). It is worth mentioning that the difference between the proportions estimated by ratios—(1) the number of unique substances in a line to the sum of unique substances for all wheat lines under study and (2) the number of unique substances in a line to the total number of biologically active substances in this line—was 3.2-fold in the control line S29: the largest difference among the seven lines (Figure 5). In all the studied lines, the contribution of phenolic compounds to the pool of unique substances was predominant (60.0–88.2%).

**Figure 6:**
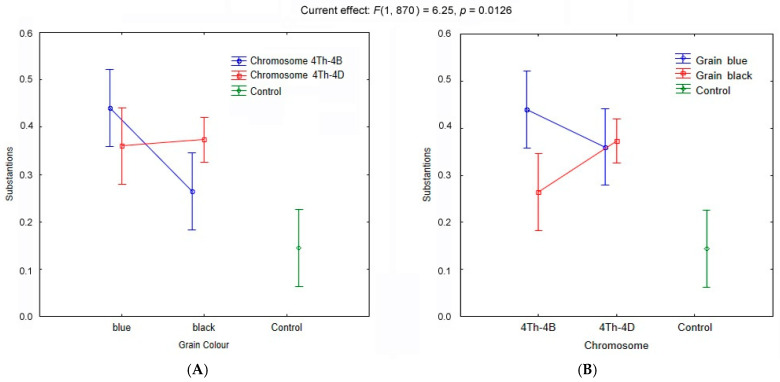
Effects of the factors “Chromosome Substitution” and “Grain Color” on diversity of chemicals in wheat grain assessed in groups of lines combined based on grain color (**A**) and substituted chromosomes (**B**), respectively, according to two-way ANOVA (Fisher’s F test). Vertical bars denote 0.95 confidence intervals.

Effects of various factors on the compounds’ diversity in the seven lines were analyzed by one-way ANOVA on ranks (Table 2). It was found that factors “Chromosome Substitution“, “Grain Color“, and “Genotype of Line” affect the diversity of the chemical compounds, but “Genotype of Parental Line/Cultivar” does not. A multiple pairwise comparison of proportions of compounds (in the total number of compounds) in the groups of the lines having substituted chromosomes 4B and 4D did not uncover any differences between the lines (*p* = 0.688, Duncan test). No such differences were revealed in the groups of lines with blue and black colors of grains (*p* = 0.229, Duncan test). Nonetheless, an effect of an interaction of two factors “Chromosome Substitution × Grain Color” on the diversity of compounds was detected (Figure 6A). In the group of the lines with substituted chromosome 4D, there were no differences between the blue- and black-grained lines (*p* = 0.807, Duncan test), whereas in the group of the lines with substituted chromosome 4B, such differences between the blue- and black-grained lines were found (*p* = 0.0023, Duncan test), with significantly lower diversity of the compounds in the latter group. In the group of blue-grained lines, lower diversity of the compounds was observed in the lines with substituted chromosome 4D, while in the black-grained lines, the effect of chromosome substitutions was opposite: higher diversity (Figure 6B).

## 3. Discussion

Successful extraction of polyphenolic compounds depends on two sequential actions: dissolution of each polyphenolic compound at the cellular level in the matrix of plant material and its diffusion into the external medium (the solvent). This is why it is difficult to develop an extraction procedure suitable for all phenolic compounds. For the extraction of phenolic compounds, various organic solvents are commonly used, such as methanol, ethanol, acetone, ethyl acetate, or combinations thereof, often with different proportions of water. Additionally, an important factor directly affecting the solubility and extraction of these compounds is pH of the extraction medium, which determines the solubility of the soluble compounds and affects the possible solubilization of the hydrolyzable fraction.

Liquid chromatography is a versatile and well-established separation technique often employed for a variety of analytical tasks and allowing the separation of fairly complex mixtures of low- and high-molecular-weight compounds. This method is also suitable for different polarities and acid-base properties of various matrices.

**Figure 7:**
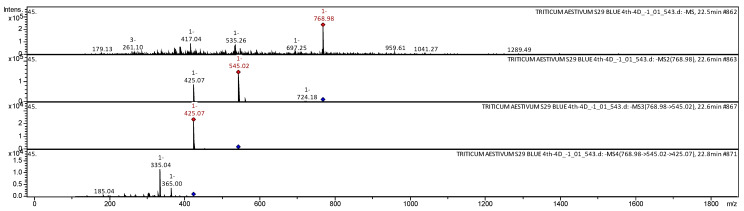
A collision-induced dissociation spectrum of apigenin 2″-*O-*sinapoyl, *C*-hexosyl, *C*-pentosyl from extracts of *T. aestivum* grains, *m*/*z* 768.98.

In this study, 125 biologically active compounds of a phenolic and nonphenolic nature were identified in differently pigmented wheat grains by HPLC coupled with Bruker Daltonics ion trap MS/MS (Table 1). Our annotation results are consistent with the extensive mass-spectrometric literature data on the wheat *T. aestivum* [27,28,29,30,31,32,33] and other plant matrices, e.g., *Passiflora incarnate* [34], *Bituminaria* [35], *Phyllostachys nigra* [36], *Carpobrotus edulis* [37], and *Vaccinium macrocarpon* [38]. For example, the collision-induced dissociation spectrum (in negative ion mode) of a flavone called apigenin 2″-*O*-sinapoyl, *C*-hexosyl, *C*-pentosyl from extracts of *T. aestivum* grains [line S29 BLUE (4Th-4D)] is given in Figure 7. The [M − H]^−^ molecular ion gave rise to three molecular ions at *m*/*z* 545.02, 724.18, and 425.07 (see Figure 7). The molecular ion with *m*/*z* 545.02 yielded one daughter ion at *m*/*z* 425.07. The molecular ion with *m*/*z* 425.07 broke up into three daughter ions with *m*/*z* 365.00, 335.04, and 185.04. It was identified in the literature about extracts from *T. aestivum* [29].

Among the identified compounds, 87 were identified in wheat grains for the first time; they are affiliated with such phenolic compounds families as anthocyanins, coumarins, dihydrochalcones, flavan-3-ols, flavanone, flavones, flavonols, hydroxybenzoic acids, hydroxycinnamic acids, isoflavone, lignans, other phenolic acids, stilbenes, and nonphenolic compounds families as alkaloids, carboxylic acids, carotenoids, diterpenoids, essential amino acids, triterpenoids, sterols, nonessential amino acids, phytohormones, purines, and thromboxane receptor.

The diversity of phytochemicals may underlie diverse biological activities of the raw material. For instance, under the common name anthocyanins, there are up to 600 individual chemicals [39]. Biological activity of some individual anthocyanins has been tested, and distinct effects on physiological processes in animals and humans (or a lack of any) have been described. Antioxidant activity of anthocyanins is reported to be dependent on structural features of the molecules such as the number of hydroxyl and methyl groups and patterns of glycosylation [40]. Among anthocyanins, the highest antioxidant activity is featured by derivatives of delphinidin and cyanidin, followed by derivatives of malvidin, peonidin, pelargonidin, and petunidin [41]. In addition, a glycoside and rutinoside of cyanidin accelerate the regeneration of rhodopsin, while the derivatives of delphinidin have no effect [42]. Anthocyanidins have been demonstrated to be better inhibitors of cell proliferation than anthocyanins [43], with delphinidin and cyanidin having the best growth-inhibitory property and pelargonidin and malvidin devoid of such effects [44,45]. From these observations, we may conclude that the more compounds are present in plant material, the wider is the expected spectrum of biological activities. Investigation of such diversity is a promising field for the development of functional food programs and for pharmacological research.

Here, we compared the diversity of compounds among colored-grain wheat lines and observed that the anthocyaninless line S29 is characterized by the lowest diversity of all the identified compounds, phenolic compounds in particular (Figure 3). The lower diversity of biologically active compounds in S29 is explained by the absence of seven families of phenolics (coumarins, flavan-3-ols, flavanones, flavonols, phenolic acids, dihydrochalcones, and stilbenes) and 12 families of nonphenolic compounds (alkaloids, anabolic steroids, carboxylic acids, carotenoids, cycloartanols, di- and triterpenoids, propionic acids, purines, sesquiterpenoid plant hormones, thromboxane receptor antagonists, and unsaturated fatty acids). These data imply that the genes of wheatgrass chromosome 4Th and chromosome fragments introgressed into 2A and 7D (including the genes regulating anthocyanin biosynthesis) are responsible for the presence of the above compounds in the grain and thus affect the diversity of biologically active substances in the wheat grain.

Although the black-grained lines contain *Pp* genes in addition to *Ba* and one may expect an increased number of biologically active compounds in these lines, there were no significant differences in the number of identified compounds between blue- and black-grained lines having chromosome 4D substituted by 4Th; moreover, a statistically significant decrease in the diversity of compounds was observed in the black-grained lines in comparison with the blue-grained lines having a chromosome 4B substitution. According to the results of our one-way ANOVA on ranks, the diversity of the chemicals is affected by such genetic factors as “Chromosome Substitution,” “Grain Color,” and “Genotype of Line,” but not “Genotype of Parental Line/Cultivar.” (Table 2) In support of these data, some differences in the chemical profile were noted among the lines with distinct substitutions of chromosomes and among lines with different colors of grains (Table S1). For example, two compounds belonging to the classes of phenolic and nonphenolic substances—selgin and a sesquiterpenoid plant hormone, respectively—were identified only in the lines with substituted chromosome 4B [S29 BLUE (4Th-4B) and S29 BLACK (4Th-4B)]. This observation suggests that this chromosome carries regulatory factors suppressing the synthesis of these compounds. Removing them by substitution of the chromosomes carrying these repressors activates the synthesis of the compounds in the substitution lines. Some common features can be found among the chemical profiles of the lines with similar chromosomes composition. Even though the sister lines of S29 are genetically related (and there is a line based on E22 that has S29 in its pedigree [46]; Figure S2), some line-specific (unique) compounds were identified (Table 1, Figure 5). They constitute unique chemical fingerprints of each line, allowing to distinguish each line from the six others. The unique compounds of each line are hardly explained by the genetic relationships among the lines but can be considered the main reason for the separation of the analyzed lines into two subclusters observed in the dendrogram and the separation of lines S29 BLACK (4Th-4D) and BW BLACK (4Th-4D), which are characterized by the highest percentage of unique compounds (Figure 4 and Figure 5).

## 4. Materials and Methods

### 4.1. Materials

The chemical profiles were analyzed in seven wheat lines with different grain colors and characterized genetic pedigrees (Table 3, Figure S2). The control group of (anthocyaninless) grains consisted of *cv*. Saratovskaya 29 (S29). Blue grains were represented by two wheat-wheatgrass substitution lines S29 BLUE(4Th-4B) and S29 BLUE (4Th-4D) developed in the S29 background but carrying *Ba* gene–containing wheatgrass chromosome 4Th, which replaced wheat chromosomes 4B and 4D, respectively [25,26]. Black grains were represented by four lines, two of them—S29 BLACK (4Th-4B) and S29 BLACK (4Th-4D)—have been developed previously in the S29 background by crossing the above-mentioned lines with purple-grained near-isogenic wheat line S29 PURPLE *Pp-D1Pp3* carrying introgressions in chromosomes 7D and 2A, onto which the dominant alleles of genes *Pp-D1* and *Pp3*, respectively, have been mapped [47,48]. Two other black-grained lines—E22 BLACK (4Th-4D) and BW BLACK (4Th-4D)— were developed in the current study by marker-assisted transfer of genes *Pp-D1+Pp3* and *Ba* from donor lines [S29 PURPLE *Pp-D1Pp3* and S29 BLUE (4Th-4D), respectively] into *cv.* Element 22 (E22) (P.A. Stolypin Omsk State Agrarian University, Omsk, Russia) and breeding line BW49880 (CIMMYT, INT, México-Veracruz, Mexico) (Figure S2).

### 4.2. Chemicals and Reagents

HPLC grade acetonitrile was purchased from Fisher Scientific (Southborough, UK), and MS grade formic acid from Sigma-Aldrich (Steinheim, Germany). Ultra-pure water was prepared by means of a SIEMENS ULTRA clear (SIEMENS Water Technologies, Munich, Germany), and all other chemicals were of analytical grade.

### 4.3. Fractional Maceration

To obtain highly concentrated extracts, fractional maceration was employed. In this technique, the total amount of an extractant (reagent grade ethyl alcohol) is divided into three parts and is sequentially applied to grains (first, the first part, then with the second and third). The infusion time for each part of the extractant was 14 days.

### 4.4. Liquid Chromatography

HPLC was performed on a Shimadzu LC-20 Prominence HPLC system (Shimadzu, Tokyo, Japan) equipped with a UV sensor and a Shodex ODP-40 4E reverse-phase column for the separation of multicomponent mixtures. The gradient elution program was as follows: from time point 0.01 min to 4.00 min, 100% A; from 4 to 60 min, 100–25% A; from 60 to 75 min, 25–0% A; then, a control wash from 75 to 120 min at 0% A. The entire HPLC analysis was carried out with an ESI detector at wavelengths of 230 and 330 nm; the temperature was set to 17 °C, and the injection volume was 1 mL.

### 4.5. MS

This analysis was performed on an ion trap amaZon SL instrument (Bruker Daltonics, Bremen, Germany) equipped with an electrospray ionization source, in negative ion mode. The following optimal parameters were found and applied: ionization source temperature 70 °C, gas flow 4 L/min, nebulizer gas (atomizer) 7.3 psi, capillary voltage 4500 V, end plate bend voltage 1500 V, fragmentation voltage 280 V, and collision energy 60 eV. The ion trap was used in the scan range *m*/*z* 100–1700 for MS and MS/MS. The capture rate was 1 spectrum/s for MS and 3 spectra/s for MS/MS. Data collection was controlled by Hystar Data Analysis 4.1 software (Bruker Daltonics, Bremen, Germany). All the measurements were performed in triplicate. The combination of both ionization modes (positive and negative) in MS full scan mode provided extra confidence of the molecular mass determination. A comprehensive table of molecular masses of the target analytes isolated from the EtOH extracts of *T. aestivum* grains was compiled by comparing the *m*/*z* values, retention times, and the fragmentation patterns with the MS/MS spectral data from the literature [28,29,31,34,49,50,51,52,53,54,55,56,57], and other sources or from searches of databases (MS2T, MassBank, and HMDB).

### 4.6. Data Analysis

A nonparametric test (Spearman’s rank correlation analysis) was performed to compare the wheat lines having different grain colors; for estimation of differences between two groups, we used the two-sided version of the test. We also carried out the Kruskal–Wallis *H* test (one-way ANOVA on ranks), the Fisher *F* test (two-way ANOVA), and multiple pairwise analysis (Duncan test) in the STATISTICA 10.0 software [58]. To visualize the obtained data, a dendrogram based on Euclidean distances was drawn by the UPGMA.

## 5. Conclusions

As shown by a number of pharmacological studies, single-component drugs cannot be sufficiently effective in the treatment of multifactorial diseases. The mixtures of biologically active compounds that possess an ability to interact with each other often turn out to be more effective against a disease as compared to individual components of the mixture. Bioactive natural products containing a wide variety of compounds are considered more attractive for the production of functional foods and pharmacological research than formulations containing only a few components. Currently, the search for raw materials with a wide variety of biologically active compounds is an urgent task. In the present study, diversity of such compounds was investigated in anthocyanin-rich wheat grains by HPLC-MS/MS. Aside from anthocyanin, the study was focused on identifying other families of compounds of a phenolic and nonphenolic nature. A total of 125 biologically active compounds were identified, and among them, 87 were found in wheat grains for the first time. Statistically significantly higher diversity of the compounds was noted in colored grains of wheat in comparison with a control line, whereas between blue- and black-grained groups of lines, no differences were found. The unique chemical profiles with line-specific compounds were determined for each anthocyanin-rich line. The results make these lines promising sources of functional-food ingredients with a wide spectrum of biological activities.

## Figures and Tables

**Table 1:** A detailed table of the biologically active substances found in the analyzed colored-grain lines of the wheat *T. aestivum*. Different color marks the presence of certain compounds in particular lines.

ID	Classes and Families of Compounds	Name	S29 Control	S29 BLUE 4Th-4B	S29 BLUE 4Th-4D	S29 BLACK 4Th-4B	S29 BLACK 4Th-4D	E22 BLACK 4Th-4D	BW BLACK 4Th-4D
	Phenolics								
1	Anthocyanin	Cyanidin 3-(2″-galloylglucoside)							yes
2		Cyanidin-3-*O*-3″,6″-*O*-Dimalonylglucoside		yes		yes		yes	
3		Cyanidin-3-*O*-glucoside					yes		
4		Malvidin 3-*O*-rutinoside					yes		
5		Malvidin 3-*O*-rutinoside-5-*O*-glucoside		yes	yes				
6		Peonidin 3-*O*-rutinoside		yes			yes		
7		Peonidin 3-rutinoside-5-glucoside							yes
8		Peonidin-3-*O*-glucoside					yes		yes
9		Petunidin	yes					yes	
10		Petunidin 3-*O*-rutinoside-5-*O*-glucoside		yes	yes				
11	Cinnamic acid derivative	Ferulic acid methyl ester					yes		
12	Hydroxycinnamic acid	1-Caffeoyl-β-d-glucose					yes		
13		1-*O*-Sinapoyl-β-d-glucose					yes		
14		Caffeic acid derivative					yes		yes
15		Caftaric acid		yes	yes			yes	yes
16		Chlorogenic acid					yes		
17		Ferulic acid	yes				yes	yes	
18	Coumarin	Fraxetin		yes					
19		Fraxetin-7-*O*-sulfate		yes					
20	Dihydrochalcone	Phlorizin							yes
21	Flavan-3-ol	Catechin [d-Catechol]		yes					yes
22		Epicatechin							yes
23		Gallocatechin [+(-)Gallocatechin]				yes			yes
24	Flavanone	Naringenin [Naringetol; Naringenine]			yes	yes		yes	yes
25	Flavone	*6-C*-hexosyl-chrysoeriol *O*-rhamnoside-*O*-hexoside	yes	yes		yes	yes	yes	
26		Acacetin *C*-glucoside methyl malonylated					yes		
27		Apigenin			yes		yes		yes
28		Apigenin 2″-*O*-sinapoyl, *C*-hexosyl, *C*-pentosyl		yes	yes				
29		Apigenin 6,8-di-*C*-pentoside		yes	yes	yes	yes		
30		Apigenin 6-*C*-deoxyhexoside-8-*C*-pentoside				yes			yes
31		Apigenin-6-*C*-β-galactosyl-8-*C*-β-glycosyl-*O*-glycuronopyranoside							yes
32		Apigenin 8-*C*-hexoside-6-*C*-pentoside	yes	yes	yes	yes	yes	yes	yes
33		Apigenin 8-*C*-pentoside-6-*C*-hexoside		yes	yes	yes	yes	yes	
34		Chrysoeriol [Chryseriol]		yes	yes	yes	yes	yes	yes
35		Chrysoeriol *C*-hexoside-*C*-pentoside		yes			yes		
36		Cirsiliol	yes						
37		Dihydroxy tetramethoxyflavone			yes				
38		Diosmetin							yes
39		Genistein *C*-glucosyl glucoside					yes		
40		Hydroxy dimethoxyflavone hexoside					yes		
41		Luteolin					yes		
42		Luteolin 8-*C*-Glucoside						yes	
43		Luteolin 8-*C*-hexoside-6*-C*-pentoside							yes
44		Luteolin 8-*C*-pentoside-6-*C*-hexoside		yes	yes	yes	yes		
45		Myricetin				yes			
46		Orientin 7-*O*-deoxyhexoside [Luteolin 8-*C*-glucoside 7-*O*-deoxyhexoside]		yes					
47		Pentahydroxy dimethoxyflavone	yes						
48		Pentahydroxy dimethoxyflavone hexoside	yes					yes	
49		Pentahydroxy trimethoxy flavone	yes			yes	yes	yes	yes
50		Tricin		yes	yes	yes	yes	yes	yes
51		Tetrahydroxy-dimethoxyflavone-hexoside		yes					
52		Trihydroxy methoxyflavone triacetate						yes	
53		Vicenin-2 [Apigenin-6,8-Di-*C*-Glucoside]		yes	yes				
54		Vitexin 2″-*O*-glucoside [Apigenin 8-*C*-glucoside 2″-*O*-glucoside]		yes			yes		
55		Vitexin 6″-*O*-glucoside [Apigenin 8-*C*-glucoside 6″-*O*-glucoside]			yes	yes			
56		Wighteone-*O*-glucoside					yes		
57	Flavonol	Ampelopsin							yes
58		Isorhamnetin					yes	yes	yes
59		Kaempferide					yes		
60		Kaempferol					yes	yes	
61		Quercetin				yes			
62		Rhamnetin I						yes	
63		Rhamnetin II					yes	yes	
64		Selgin		yes		yes			
65		Taxifolin-3-*O*-glucoside		yes	yes	yes		yes	
66		Taxifolin-*O*-pentoside					yes	yes	yes
67	Gallotannin	β-Glucogallin [1-*O*-Galloyl-β-d-Glucose]	yes	yes	yes		yes	yes	
68	Hydroxybenzoic acid	4-Hydroxybenzoic acid		yes					
69		*Cis*-salvianolic acid J		yes				yes	yes
70		Hydroxy methoxy dimethylbenzoic acid		yes					yes
71		Salvianolic acid D	yes	yes	yes	yes		yes	yes
72		Salvianolic acid F		yes					
73		Salvianolic acid G			yes			yes	yes
74	Lignan	Dimethyl-secoisolariciresinol			yes				
75		Hinokinin		yes	yes	yes		yes	
76		Pinoresinol							yes
77		Podophyllotoxin [Podofilox; Condylox; Condyline; Podophyllinic acid lactone]					yes		
78		Syringaresinol	yes	yes	yes		yes		yes
79	Phenolic acid	1-*O*-caffeoyl-5-*O*-feruloylquinic acid		yes	yes	yes	yes		
80		4-*O*-Caffeoyl-5-*O*-*p*-coumaroylquinic acid							yes
81		Feruloyl sulfate		yes					
82	Phenolic glucoside	Gallic acid hexoside	yes						
83	Stilbene	Pinosylvin			yes				
84		Polydatin [Piceid; *trans*-Piceid]						yes	
85		Resveratrol					yes		
	**Others**								
86	Alpha, omega-dicarboxylic acid	Undecanedioic acid					yes		yes
87	Carboxylic acid	Myristoleic acid [*Cis*-9-Tetradecanoic acid]		yes	yes	yes		yes	yes
88	Higher-molecular-weight carboxylic acid	11-Hydroperoxy-octadecatrienoic acid					yes		
89		9,10-Dihydroxy-8-oxooctadec-12-enoic acid		yes	yes	yes			
90		Dihydroxy docosanoic acid		yes	yes	yes	yes	yes	yes
91		Docosenoic acid [2-Docosenoic acid]							yes
92		Hydroxy methoxy dimethylbenzoic acid					yes		
93		Pentacosenoic acid	yes	yes	yes	yes	yes	yes	yes
94		Salvianic acid C			yes	yes	yes		yes
95	Anabolic steroid	Vebonol		yes	yes		yes		yes
96	Cycloartanol [Steroids]	Cyclopassifloic acid glucoside			yes			yes	
97	Carotenoid	(3*S*, 3′*S*, all-*E*)-zeaxanthin [Zeaxanthin; (3*S*,3′*S*)-Zeaxanthin]		yes	yes			yes	yes
98		Cryptoxanthin [β-cryptoxanthin]		yes			yes		
99	Diterpenoid	Isocryptotanshinone II		yes	yes				
100		Tanshinone IIB							yes
101	Pentacyclic diterpenoid	β-Amyrin [β-Amyrenol; Amyrin]						yes	
102		Gibberellic acid			yes				
103	Triterpenic acid	Betunolic acid							yes
104		Ursolic acid							yes
105	Triterpenoid	Squalene		yes					
106		Uvaol				yes			
107	Essential amino acid	l-Histidine		yes			yes		
108		l-Tryptophan [Tryptophan; (*S*)-Tryptophan]	yes	yes	yes	yes	yes	yes	
109		l-Valine			yes				
110	Nonessential amino acid	Tyrosine	yes						
111	Indole sesquiterpene alkaloid	Sespendole			yes		yes		
112	Isoquinoline alkaloid	Berberine [Berberin; Umbelletine; Berbericine]					yes		yes
113	Phytohormone	GA8-hexose gibberellin	yes	yes	yes		yes		yes
114	Sesquiterpenoid plant hormone	Abscisic acid [Dormin; Abscisin II; (*S*)-(+)-Abscisic acid]		yes		yes			
115	Propionic acid	Ketoprofen [Orudis; 2-(3-Benzoylphenyl)Propionic acid]		yes	yes	yes			yes
116	Purine	Adenosine		yes	yes		yes		
117	Phytosterol	Ergosterol [Provitamin D2; Ergosterin]	yes	yes	yes	yes	yes	yes	yes
118	Sterol	Avenasterol	yes	yes	yes	yes	yes	yes	yes
119		β-Sitostenone [Stigmast-4-En-3-One; Sitostenone]			yes		yes	yes	
120		β-Sitosterin [β-Sitosterol]		yes			yes	yes	
121		Campestenone		yes	yes	yes	yes		yes
122		Fucosterol		yes		yes			yes
123		Oxo-hydroxy sitosterol		yes					
124	Thromboxane receptor antagonist	Vapiprost		yes	yes				
125	Unsaturated fatty acid	Hexadecatrienoic acid [Hexadeca-2,4,6-trienoic acid]							yes

**Table 2:** Effects of various factors on the diversity of phenolic and nonphenolic compounds in the seven wheat lines according to the Kruskal-Wallis *H* test (i.e., one-way ANOVA on ranks; *df*: degrees of freedom).

Factor	Group	Group Size	*df*	Sum of Ranks	Mean Rank	*H* Criterion	*p* Value	Significant Result
Chromosome Substitution	4Th-4B	2	2	111,625.0	446.5	23.58	0.00001	yes
	4Th-4D	4		227,187.5	454.4			
	Control	1		44,437.5	355.5			
Genotype of Parental Line/Cultivar	BW	1	2	57,562.5	460.5	2.26	0.322	no
	E22	1		52,750.0	422.0			
	S29	5		272,937.5	436.7			
Grain Color	Black grains	4	2	443.8750	443.8	25.52	0.00001	yes
	Blue grains	2		116,875.0	467.5			
	Control	1		44,437.5	355.5			
Genotype of Line	S29 BLUE (4Th-4B)	1	6	60,625.0	485.0	38.29	0.00001	yes
	S29 BLUE (4Th-4D)	1		56,250.0	450.0			
	S29 BLACK (4Th-4B)	1		51,000.0	408.0			
	S29 BLACK (4Th-4D)	1		60,625.0	485.0			
	E22 BLACK (4Th-4D)	1		57,562.5	460.5			
	BW BLACK (4Th-4D)	1		52,750.0	422.0			
	Control	1		44,437.5	355.5			

**Table 3:** Genetic characteristics of the wheat lines used in this study.

Genotype	Recurrent Parent	Grain Color	*Ba*	*Pp-D1 + Pp3*	Substituted Chromosome	References
S29 BLUE(4Th-4D)	S29	blue	+	-	4D	[25]
S29 BLUE(4Th-4B)	S29	blue	+	-	4B	[26]
S29 BLACK(4Th-4B)	S29	black	+	+	4B	
S29 BLACK(4Th-4D)	S29	black	+	+	4D	
BW BLACK(4Th-4D)	BW49880	black	+	+	4D	Figure S2
E22 BLACK(4Th-4D)	Element 22	black	+	+	4D	

## Data Availability

The data presented in this study are available on request from the corresponding authors.

## Supplementary Materials

The following are available online, Figure S1: Dendrograms for seven *T. aestivum* lines. The trees were built using the UPGMA and Euclidean distance from data on different groups of chemicals, Figure S2: The breeding scheme for the development of the blue- and black-grained wheat lines used in this study, Table S1: The presence of biologically active compounds in the wheat lines grouped by chromosome substitution, grain color, or both.

## Appendix A

**Table A1:** The list of compounds identified in EtOH extracts of *T. aestivum* grains.

ID	Identified Compound	Molecular Formula	Calculated Mass	Observed Mass		Fragment Ions, *m*/*z*	References
				[M − H]^–^	[M + H]^+^		
**Anthocyanin**							
1	Cyanidin 3-(2″-galloylglucoside)	C_28_H_25_O_15+_	601.4891		602	592; 556; 429; 349; 287; 231	[59]
2	Cyanidin-3-*O*-3″,6″-*O*-Dimalonylglucoside	C_27_H_25_O_17_	621.4772		622	612,395, 328, 287, 221	[31]
3	Cyanidin-3-*O*-glucoside [Cyanidin 3-*O*-β-d-Glucoside; Kuromarin]	C_21_H_21_O_11_+	449.3848		450	287; 213; 169; 115	[31,54,60]
4	Malvidin 3-*O*-rutinoside	C_29_H_35_O_16_	639.5786		640	493; 331; 315	[31,61]
5	Malvidin 3-*O*-rutinoside-5-*O*-glucoside	C_35_H_45_O_21_	801.7192		802	639; 493; 331	[31,61]
6	Peonidin 3-*O*-rutinoside	C_28_H_33_O_15_	609.5526		610	463; 343; 301; 285; 258	[31,59,62]
7	Peonidin 3-rutinoside-5-glucoside	C_34_H_43_O_20_	771.6932		772	705; 463; 367; 301	[31]
8	Peonidin-3-*O*-glucoside	C_22_H_23_O_11 +_	463.4114		464	301; 445; 286; 258	[31,59,63]
9	Petunidin	C_16_H_13_O_7_	317.2702		318	300; 256; 238; 198	[31,61]
10	Petunidin 3-*O*-rutinoside-5-*O*-glucoside	C_34_H_43_O_21_	787.6926		788	625; 479; 317	[31,61]
**Cinnamic Acid Derivative**							
11	Ferulic acid methyl ester	C_11_H_12_O_4_	208.2106	207		179; 135	[64]
**Hydroxycinnamic Acid**							
12	1-Caffeoyl-β-d-glucose [Caffeic acid-glucoside]	C_15_H_18_O_9_	342.298	341		161; 143	[34,63]
13	1-*O*-Sinapoyl-β-d-glucose	C_17_H_22_O_10_	386.3576		387	205	[63]
14	Caffeic acid derivative	C_16_H_18_O_9_Na	377.2985	377		341; 178; 143	[65]
15	Caftaric acid [*Cis*-Caftaric acid; 2-Caffeoyl-l-Tartaric acid]	C_13_H_12_O_9_	312.23	311		267; 293; 249; 193; 183; 167	[37,49,63,66]
16	Chlorogenic acid [3-*O*-Caffeoylquinic acid]	C_16_H_18_O_9_	354.3087		355	337; 319; 301; 239; 222; 227	[54,67]
17	Ferulic acid	C_10_H_10_O_4_	194.184		195	137	[54,68]
**Coumarin**							
18	Fraxetin	C_10_H_8_O_5_	208.1675	207		179; 161; 135; 117	[69,70,71]
19	Fraxetin-7-*O*-sulfate	C_10_H_8_O_8_S	288.2307	287		207; 163; 119	[69]
**Dihydrochalcone**							
20	Phlorizin [Phloridzin; Phlorizoside; Floridzin; Phloretin 2′-Glucoside]	C_21_H_24_O_10_	436.4093		437	275; 257; 203; 173; 150	[37,53,55,72]
**Flavan-3-ol**							
21	Catechin [d-Catechol]	C_15_H_14_O_6_	290.2681		291	245; 203; 145	[53,55,56,66,68,73,74,75,76]
22	Epicatechin	C_15_H_14_O_6_	290.2681		291	273; 255; 213; 147; 127	[37,53,63,68,75,76,77]
23	Gallocatechin [+(−) Gallocatechin]	C_15_H_14_O_7_	306.2675	305		287; 249; 205; 151; 138; 125	[37,66,73]
**Flavanone**							
24	Naringenin [Naringetol]	C_15_H_12_O_5_	272.5228		273	156; 191; 112	[53,57,63,73,78]
**Flavone**							
25	6-*C*-hexosyl-chrysoeriol *O*-rhamnoside-*O*-hexoside	C_33_H_38_O_21_	770.6422		771	753; 704; 687; 585; 529; 499; 427; 422; 385; 337; 207	[27]
26	Acacetin *C*-glucoside methylmalonylated	C_26_H_26_O_13_	546.4758		547	529; 511; 427; 301; 253; 172	[79]
27	Apigenin	C_15_H_10_O_5_	270.2369		271	252; 239; 226; 211	[34]
28	Apigenin 2″-*O*-sinapoyl, *C*-hexosyl, *C*-pentosyl	C_37_H_38_O_18_	770.6868	768		545; 425; 365; 335; 185	[29]
29	Apigenin 6,8-di-*C*-pentoside	C_25_H_26_O_13_	534.4661		535	499; 481; 415; 409; 307; 291	[34,49,80]
30	Apigenin 6-*C*-deoxyhexoside-8-*C*-pentoside	C_26_H_28_O_13_	548.4927		549	531; 465; 369; 319; 248	[34]
31	Apigenin-6-*C*-β-galactosyl-8-*C*-β-glycosyl-*O*-glycuronopyranoside	C_33_H_38_O_21_	770.6422		771	679; 651; 561; 511; 457; 367; 313; 297; 267; 249; 215; 207; 177; 121	[28]
32	Apigenin 8-*C*-hexoside-6-*C*-pentoside	C_26_H_28_O_14_	564.4921		565	547; 529; 511; 481; 427; 349; 325; 313	[28,29,35]
33	Apigenin 8-*C*-pentoside-6-*C*-hexoside	C_26_H_28_O_14_	564.4921		565	547; 529; 511; 427; 391; 325; 291;	[28,29,35]
34	Chrysoeriol	C_16_H_12_O_6_	300.2629		301	286; 258; 229	[33,60,81]
35	Chrysoeriol *C*-hexoside-*C*-pentoside	C_27_H_30_O_15_	594.5181		595	577; 558; 499; 481; 427; 379; 327; 287	[27,29,32,35]
36	Cirsiliol	C_17_H_14_O_7_	330.2889	329		229; 211; 171; 155	[52]
37	Dihydroxy tetramethoxyflavanone	C_19_H_20_O_8_	376.3573		377	361; 323; 265; 179	[37]
38	Diosmetin [Luteolin 4′-Methyl Ether; Salinigricoflavonol]	C_16_H_12_O_6_	300.2629		301	286; 258; 177; 138	[81,82,83]
39	Genistein *C*-glucosylglucoside	C_27_H_30_O_15_	594.5181		595	577; 529; 457; 427; 302	[79]
40	Hydroxy dimethoxyflavone hexoside	C_23_H_24_O_10_	460.4307		461	301; 286; 258; 243	[37]
41	Luteolin	C_15_H_10_O_6_	286.2363	285		199; 151	[33,36,50,52,73,84]
42	Luteolin 8-*C*-Glucoside [Orientin; Orientin (Flavone); Lutexin]	C_21_H_20_O_11_	448.3769		449	431; 413; 356; 333; 290; 267; 233; 227	[28,36,49,55]
43	Luteolin 8-*C*-hexoside-6-*C*-pentoside	C_26_H_28_O_15_	580.4915		581	563; 515; 485; 413; 377; 342; 205	[27,29,32,35]
44	Luteolin 8-*C*-pentoside-6-*C*-hexoside	C_26_H_28_O_15_	580.4915		581	563; 528; 515; 496; 443; 413; 341; 335; 323; 181	[28,29,33]
45	Myricetin	C_15_H_10_O_8_	318.2351	317		273; 260; 238	[37,38,63,73,85]
46	Orientin 7-*O*-deoxyhexoside [Luteolin 8-*C*-glucoside 7-*O*-deoxyhexoside]	C_27_H_30_O_15_	594.5181		595	577; 528; 510; 438; 427; 325	[34]
47	Pentahydroxy dimethoxyflavone	C_17_H_14_O_9_	362.2877		363	345; 326; 247; 201; 155	[37]
48	Pentahydroxy dimethoxyflavone hexoside	C_23_H_24_O_14_	524.4283		525	463; 363; 257	[37]
49	Pentahydroxy trimethoxy flavone	C_18_H_16_O_10_	392.3136		393	375; 357; 328; 269; 230; 218	[37]
50	Tricin	C_17_H_14_O_7_	330.2889		331	315; 287; 285; 270; 229	[28,33,36,54]
51	Tetrahydroxy-dimethoxyflavone-hexoside [Syringetin-hexoside; dimethyl-myricetin-hexoside]	C_23_H_24_O_13_	508.4289		509	347; 329; 316; 265; 185; 181	[38,49,80]
52	Trihydroxy methoxyflavone triacetate	C_18_H_18_O_9_	378.3301		379	361; 321; 287; 234; 223; 167	[37]
53	Vicenin-2 [Apigenin-6,8-Di-*C*-Glucoside]	C_27_H_30_O_15_	594.5181		595	577; 559; 541; 529; 523; 499; 469; 439; 427; 391	[28,29,30,34,35,50,55]
54	Vitexin 2″-*O*-glucoside [Apigenin 8-*C*-glucoside 2″-*O*-glucoside]	C_27_H_30_O_15_	594.5181		595	577; 541; 457; 288	[34]
55	Vitexin 6″-*O*-glucoside [Apigenin 8-*C*-glucoside 6″-*O*-glucoside]	C_27_H_30_O_15_	594.5181		595	577; 559; 528; 511; 499; 493; 487; 445; 427	[34]
56	Wighteone*-O-g*lucoside	C_26_H_28_O_10_	500.4945		501	339; 262; 185; 167	[79]
**Flavonol**							
57	Ampelopsin [Dihydromyricetin; Ampeloptin]	C_15_H_12_O_8_	320.251		321	303; 285; 163	[86,87]
58	Isorhamnetin [Isorhamnetol; Quercetin 3′-Methyl ether; 3-Methylquercetin]	C_16_H_12_O_7_	316.2623	315		300; 272; 243; 145	[38,53]
59	Kaempferide	C_16_H_12_O_6_	300.2629		301	286; 258; 229; 174; 153	[52,88]
60	Kaempferol	C_15_H_10_O_6_	286.2363		287	268; 214; 196; 160; 123	[52,63,73,89]
61	Quercetin	C_15_H_10_O_7_	302.2357	301		283; 255; 227	[38,53,54,57,63,68,73,76]
62	Rhamnetin I [β-Rhamnocitrin; Quercetin 7-Methyl ether]	C_16_H_12_O_7_	316.2623		317	255; 197; 139; 122	[86]
63	Rhamnetin II	C_16_H_12_O_7_	316.2623		317	256; 121; 228; 111	[86]
64	Selgin [Selagin; 3′-*O*-Methyltricetin; Tricetin ′3-*O*-methyl ether]	C_16_H_12_O_7_	316.2623		317	315; 256; 161	[90]
65	Taxifolin-3-*O*-glucoside	C_21_H_22_O_12_	466.3922		467	449; 373; 258; 199; 177	[63]
66	Taxifolin-*O*-pentoside [Dihydroquercetin pentoside]	C_20_H_20_O_11_	436.371		437	303; 259; 177; 169	[37]
**Gallotannin**							
67	β-Glucogallin [1-*O*-Galloyl-β-d-Glucose; Galloyl glucose]	C_13_H_16_O_10_	332.2601	331		313; 295; 277; 171; 140; 127	[53,91]
**Hydroxybenzoic Acid**							
68	4-Hydroxybenzoic acid [PHBA; Benzoic acid]	C_7_H_6_O_3_	138.1207		139	137; 121	[35,49,63,74]
69	*Cis*-salvianolic acid J	C_27_H_22_O_12_	538.4564		539	523; 481; 393; 360; 319; 247; 204; 191; 120	[81]
70	Hydroxy methoxy dimethylbenzoic acid	C_10_H_12_O_4_	196.1999		197	179; 160; 133	[37]
71	Salvianolic acid D	C_20_H_18_O_10_	418.3509	417		373; 329; 287	[49,92]
72	Salvianolic acid F	C_17_H_14_O_6_	314.2895	313		295; 277; 223; 171; 155	[49,92]
73	Salvianolic acid G	C_18_H_12_O_7_	340.2837		341	323; 260; 199; 168	[81,92]
**Lignan**							
74	Dimethyl-secoisolariciresinol	C_22_H_30_O_6_	390.4700		391	355; 336; 308; 218; 149	[93]
75	Hinokinin	C_20_H_18_O_6_	354.3533		355	336; 318; 300; 207; 181; 177	[28,30,54]
76	Pinoresinol	C_20_H_22_O_6_	358.3851	357		339; 311; 267; 213; 197; 171; 155; 139	[28,30,34]
77	Podophyllotoxin [Podofilox; Condylox; Condyline; Podophyllinic acid lactone]	C_22_H_22_O_8_	414.4053		415	397; 379; 310; 275; 250; 182	[93]
78	Syringaresinol	C_22_H_26_O_8_	418.4436		419	357; 327; 275; 185; 158	[54]
**Phenolic Acid**							
79	1-*O*-caffeoyl-5-*O*-feruloylquinic acid	C_26_H_26_O_12_	530.4774		531	513; 415; 337; 195; 176; 115	[52]
80	4-*O*-Caffeoyl-5-*O*-*p*-coumaroylquinic acid	C_25_H_24_O_11_	500.4515		501	339; 244; 189; 140	[55,84]
81	Feruloyl sulfate	C_10_H_10_O_7_S	274.2472	273		193; 192; 149	[94]
82	Gallic acid hexoside	C_13_H_16_O_10_	332.2601		333	242; 212; 182; 159	[95]
**Stilbene**							
83	Pinosylvin [3,5-Stilbenediol; *Trans*-3,5-Dihydroxystilbene]	C_14_H_12_O_2_	212.2439		213	197; 183; 166; 124	[96]
84	Polydatin [Piceid; *trans*-Piceid]	C_20_H_22_O_8_	390.3839		391	355; 333; 265; 227; 209; 145	[73,77]
85	Resveratrol [*trans*-Resveratrol; 3,4′,5-Trihydroxystilbene; Stilbentriol]	C_14_H_12_O_3_	228.2433		229	228; 142; 114	[37,73]
**Other Compounds**							
86	Undecanedioic acid	C_11_H_20_O_4_	216.2741		217	173; 157; 142; 118; 115	[34]
87	Myristoleic acid [*Cis*-9-Tetradecanoic acid]	C_14_H_26_O_2_	226.3550		227	209; 138; 127; 110	[34]
88	11-Hydroperoxy-octadecatrienoic acid	C_18_H_30_O_4_	310.4284	309		291; 209; 207; 125	[97]
89	9,10-Dihydroxy-8-oxooctadec-12-enoic acid [oxo-DHODE; oxo-Dihydroxy-octadecenoic acid]	C_18_H_32_O_5_	328.4437	327		229; 211; 171; 135; 125	[35,36]
90	Dihydroxy docosanoic acid	C_22_H_44_O_4_	372.5824	371		327; 297; 282; 251; 187; 125	[34]
91	Docosenoic acid [2-Docosenoic acid])	C_22_H_42_O_2_	338.5677		339	322; 295; 256; 215; 163	[34]
92	Hydroxy methoxy dimethylbenzoic acid	C_10_H_12_O_4_	196.1999	195		177; 129	[34]
93	Pentacosenoic acid	C_25_H_48_O_2_	380.6474		381	363; 293; 173; 135	[34]
94	Salvianic acid C	C_18_H_18_O_9_	378.3301		379	361; 343; 335; 326; 247; 237; 205; 151; 129	[92]
95	Vebonol	C_30_H_44_O_3_	452.6686		453	435; 336; 209	[86]
96	Cyclopassifloic acid glucoside	C_37_H_62_O_12_	698.8810		699	537; 421; 348; 203	[31]
97	(3*S*, 3′*S*, all-*E*)-zeaxanthin [Zeaxanthin; (3*S*,3′*S*)-Zeaxanthin]	C_40_H_56_O_2_	568.8714		569	551; 375; 329; 279; 235; 210; 153	[51]
98	Cryptoxanthin [β-cryptoxanthin]	C_40_H_56_O	552.872		553	461; 337; 199	[51]
99	Isocryptotanshinone II	C_19_H_20_O_3_	296.3603		297	279; 149; 146	[98]
100	Tanshinone IIB [(*S*)-6-(Hydroxymethyl)-1,6-Dimethyl-6,7,8,9-Tetrahydrophenanthro [1,2-*B*]Furan-10,11-Dione]	C_19_H_18_O_4_	310.3438	309		291; 273; 251; 235; 209; 207; 122	[98]
101	β-Amyrin [β-Amyrenol; Amyrin; Olean-12-en-3β-ol]	C_30_H_50_O	426.7174		427	409; 391; 373; 292; 269; 240; 190; 145; 137	[34]
102	Gibberellic acid	C_19_H_22_O_6_	346.3744		347	301; 282; 263; 242; 201; 185; 139	[99]
103	Betunolic acid	C_30_H_46_O_3_	454.3446		455	436; 355; 236; 226	[86]
104	Ursolic acid	C_30_H_48_O_3_	456.7003		457	439; 263; 177; 145	[52,56,81,100]
105	Squalene (*Trans*-Squalene; Spinacene; Supraene)	C_30_H_50_	410.718		411	235; 218; 177; 147	[101,102]
106	Uvaol	C_30_H_50_O_2_	442.7168		443	425; 407; 315; 304; 287; 230; 154; 137	[34]
107	l-Histidine	C_6_H_9_N_3_O_2_	155.1546		156	…	[103]
108	l-Tryptophan [Tryptophan; (*S*)-Tryptophan]	C_11_H_12_N_2_O_2_	204.2252		205	188; 146; 118	[34,91,104]
109	l-Valine	C_5_H_11_NO_2_	117.1463		118	…	[103]
110	Tyrosine [(2*S*)-2-Amino-3-(4-Hydroxyphnyl) Propanoic acid]	C_9_H_11_NO_3_	181.19		182	155; 127; 116	[104]
111	Sespendole	C_33_H_45_NO_4_	519.7147		520	184; 125	[86]
112	Berberine [Berberin; Umbelletine; Berbericine]	C_20_H_18_NO_4_	336.3612		337	320; 303; 207; 206; 115	[105]
113	GA8-hexose gibberellin	C_25_H_34_O_12_	526.5303		527	365; 305; 275; 245; 203; 143	[91]
114	Abscisic acid [Dormin; Abscisin II; (*S*)-(+)-Abscisic acid]	C_15_H_20_O_4_	264.3169		265	247; 122	[99]
115	Ketoprofen [Orudis; 2-(3-Benzoylphenyl) Propionic acid; Profenid]	C_16_H_14_O_3_	254.2806	253		209; 191; 165; 121	[106]
116	Adenosine	C_10_H_13_N_5_O_4_	267.2413		268	136	[103]
117	Ergosterol [Provitamin D2; Ergosterin]	C_28_H_44_O	396.6484		397	379; 361; 309; 282; 239; 189; 125	[34]
118	Avenasterol [Delta7-Avenasterol; 7-Dehydroavenasterol]	C_29_H_48_O	412.6908		413	395; 376; 358; 336; 325; 271; 269; 251; 225; 224; 201; 165; 159; 124	[34]
119	β-Sitostenone [Stigmast-4-En-3-One; Sitostenone]	C_29_H_48_O	412.6908		413	493; 375; 358; 269; 261; 235; 152; 147	[34]
120	β-Sitosterin [β-Sitosterol]	C_29_H_50_O	414.7067		415	395; 377; 297; 268; 213; 163 133;	[37,100]
121	Campestenone	C_28_H_46_O	398.6642		399	337; 319; 311; 266; 239; 189; 182; 127	[34]
122	Fucosterol [Fucostein; *Trans*-24-Ethylidenecholesterol]	C_29_H_48_O	412.6908		413	395; 375; 355; 340; 303; 267; 201; 195; 167; 121	[34]
123	Oxo-hydroxy sitosterol	C_29_H_48_O_3_	444.6896		445	427; 385; 319; 205; 165; 164; 137	[34]
124	Vapiprost	C_30_H_39_NO_4_	477.6350		478	337; 121; 263	[86]
125	Hexadecatrienoic acid [Hexadeca-2,4,6-trienoic acid]	C_16_H_26_O_2_	250.3764		251	233; 204; 147	[34]
