# Supplementary material for: Phytochemical Analysis of Phenolics, Sterols, and Terpenes in Colored Wheat Grains by Liquid Chromatography with Tandem Mass Spectrometry
Source: Molecules. 2021 Sep 14;26(18):5580. doi: 10.3390/molecules26185580 (PMC8469967; doi:10.3390/molecules26185580)
Supplement: Supplementary file 1 [file molecules-26-05580-s001.zip › molecules-1362350-supplementary.pdf]

molecules

MDPI

Supplementary Materials

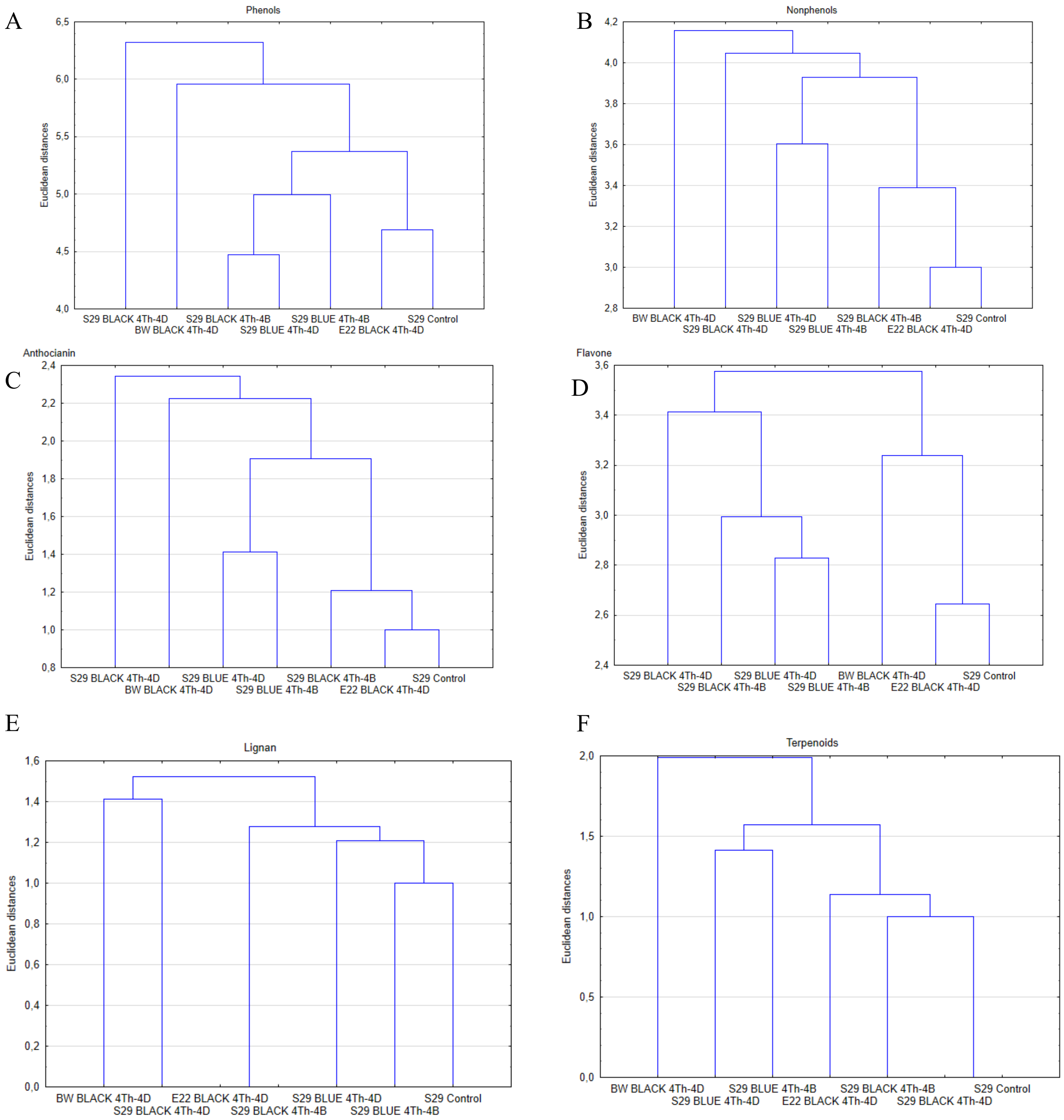

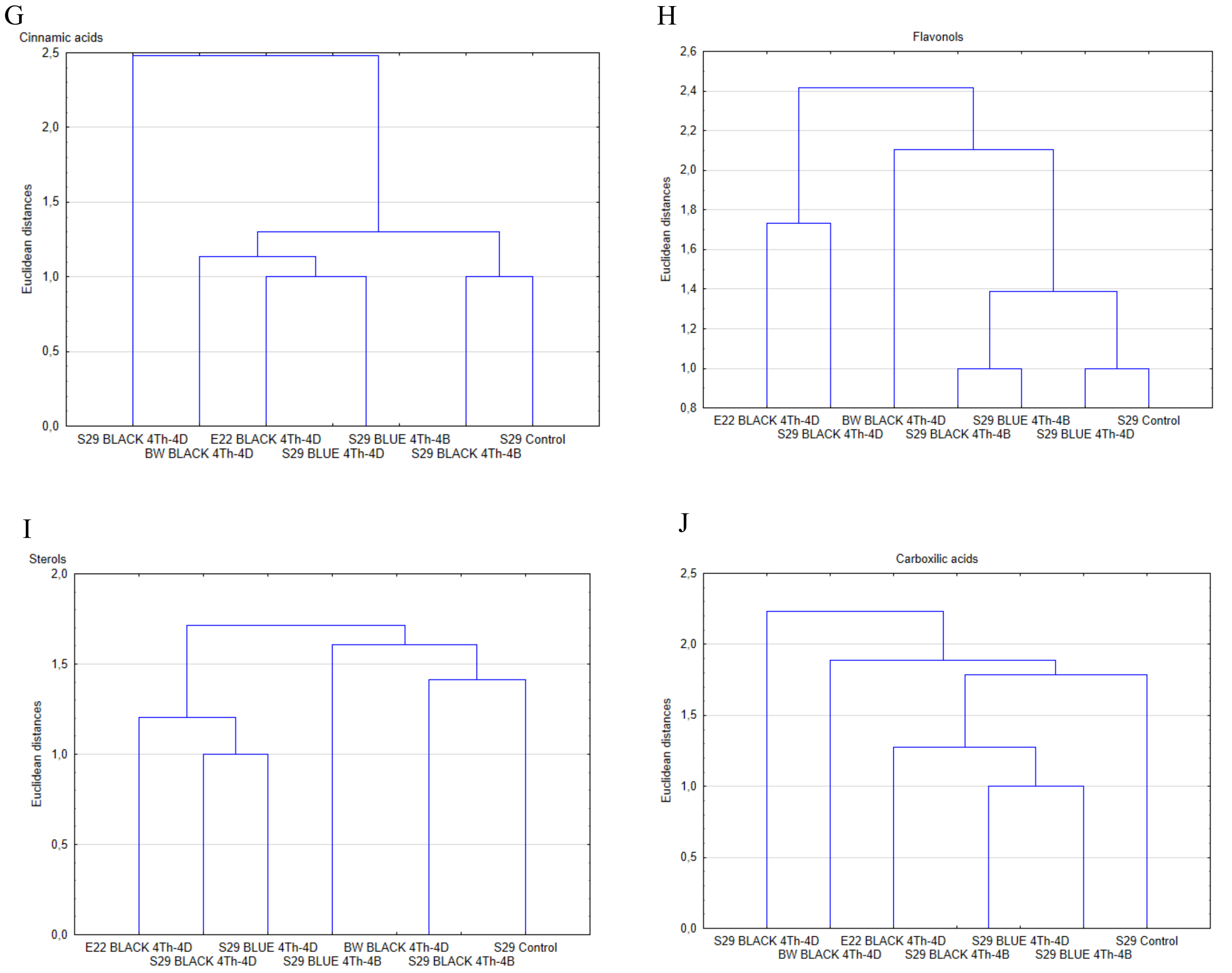

Figure S1. Dendrograms for seven *T. aestivum* lines. The trees were built using the UPGMA and Euclidean distance from data on phenolic (A), nonphenolic (B) substances, anthocyanins (C), flavones (D), lignins (E), terpenoids (F), cinnamic acids (G), flavonols (H), sterols (I), and carboxyl acids (J).

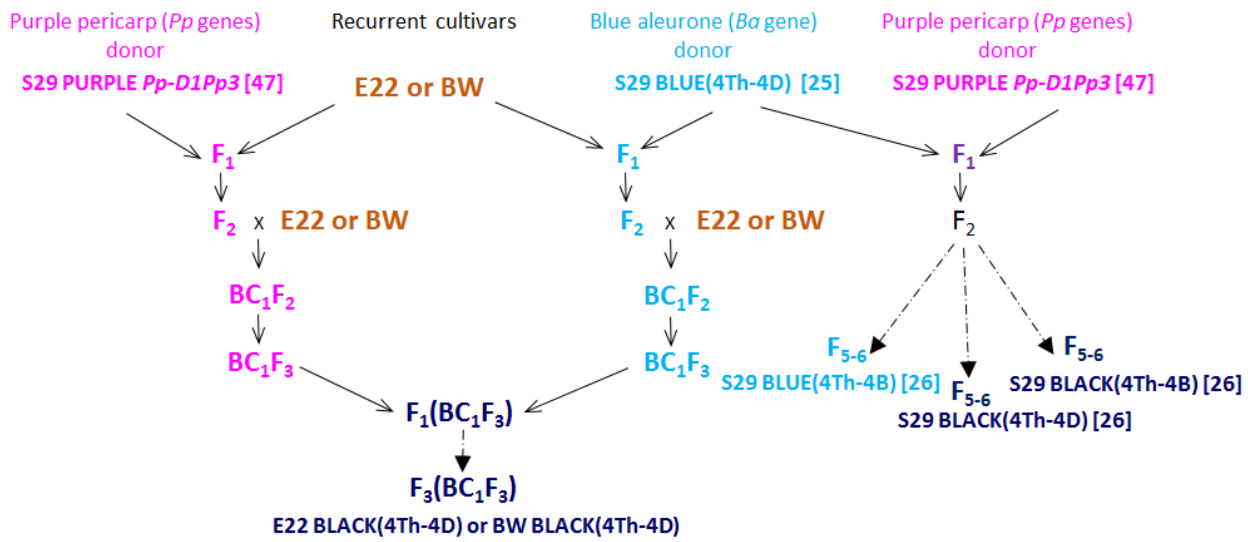

Figure S2. The breeding scheme for the development of the blue- and black-grained wheat lines used in this study. Abbreviations: BW, BW49880; E22, Element 22; S29, Saratovskaya 29.

Table S1. The presence of biologically active compounds in the wheat lines grouped by chromosome substitution, grain color, or both.

Class	Family of Compounds	Identification	S29	S29 BLACK 4Th-4B	S29 BLUE 4Th-4B	S29 BLUE 4Th-4D	S29 BLACK 4Th-4D	E22 BLACK 4Th-4D	BW BLACK 4Th-4D
The compounds of the lines with chromosome 4B substituted									
PHENOLICS	Flavonol	Selgin		1	1				
OTHERS	Sesquiterpenoid plant hormone	Abscisic acid [Dormin; Abscisin II; (S)-(+)-Abscisic acid]		1	1				
The compounds of the lines with chromosome 4D substituted									
PHENOLICS	Anthocyanin	Peonidin-3-*O*-glucoside					1		1
	Hydroxycinnamic acid	Caffeic acid derivative					1		1
	Flavone	Apigenin				1	1		1
	Flavonol	Isorhamnetin					1	1	1
		Kaempferol					1	1	
		Rhamnetin II					1	1	
		Taxifolin-*O*-pentoside					1	1	1
	Hydroxybenzoic acid	Salvianolic acid G				1		1	1
OTHERS	Alpha, omega-dicarboxylic acid	Undecanedioic acid					1		1
	Cycloartanol [Steroids]	Cyclopassifloic acid glucoside				1		1	
	Indole sesquiterpene alkaloid	Sespendole				1	1		
	Isoquinoline alkaloid	Berberine [Berberin; Umbelletine; Berbericine]					1		1
	Sterol	β-Sitostenone [Stigmast-4-En-3-One; Sitostenone]				1	1	1	
The compounds of the lines with blue color of grain									
PHENOLICS	Anthocyanin	Malvidin 3-*O*-rutinoside-5-*O*-glucoside			1	1			
		Petunidin 3-*O*-rutinoside-5-*O*-glucoside			1	1			
	Flavone	Apigenin 2''-*O*-sinapoyl, *C*-hexosyl, *C*-pentosyl			1	1			
	Glycosylated flavone	Vicenin-2 [Apigenin-6,8-Di-*C*-Glucoside]			1	1			
OTHERS	Diterpenoid	Isocryptotanshinone II			1	1			
	Thromboxane receptor antagonist	Vapiprost			1	1			
The compounds of the lines with black color of grain									
PHENOLICS	Flavonol	Isorhamnetin					1	1	1
		Taxifolin-*O*-pentoside					1	1	1
